# YTH StreetConnect: Development and Usability of a Mobile App for Homeless and Unstably Housed Youth

**DOI:** 10.2196/mhealth.5168

**Published:** 2016-07-14

**Authors:** Bhupendra Sheoran, Cara Lynn Silva, James Elliot Lykens, Londiwe Gamedze, Samantha Williams, Jessie VanNess Ford, Melissa A Habel

**Affiliations:** ^1^ YTH Oakland, CA United States; ^2^ Centers for Disease Control and Prevention Atlanta, GA United States; ^3^ New York University New York, NY United States

**Keywords:** mHealth, homelessness, youth, STD, sexually transmitted diseases, mobile app

## Abstract

**Background:**

Homeless and unstably housed (H/UH) youth are disproportionately affected by sexual health issues, including human immunodeficiency virus/sexually transmitted diseases, teen pregnancy, and dating violence, and are at a higher risk for poor mental health and underutilization of services. Research suggests that linking health care to H/UH adolescents might help improve their continuity of care, with most preferring to access health care information via the Internet. YTH StreetConnect is a dual-purpose mobile app that helps H/UH youth access health and vital services in Santa Clara County, CA, USA. We developed YTH StreetConnect PRO in parallel with the youth app as a companion tablet app for providers who serve H/UH youth.

**Objective:**

The objective of our study was to develop a mobile app to support H/UH youth and their providers in accessing health and vital resources, and to conduct usability and feasibility testing of the app among H/UH youth and technical consultants with local expertise in serving H/UH youth.

**Methods:**

Formative research included a literature review on H/UH youths’ mobile phone and Internet usage. In January 2015, we conducted interviews with medical and service providers of H/UH youth. Usability and feasibility testing were done with target audiences. Additionally, we conducted focus groups with youth regarding the app’s youth friendliness, accessibility, and usefulness.

**Results:**

H/UH youth and their providers noted the app’s functionality, youth friendliness, and resources. Usability testing proposed improvements to the app, including visual updates to the user interface, map icons, new underrepresented resource categories, and the addition of a peer rating system. Limitations included a small sample size among H/UH youth and providers and a single site for the study (Santa Clara County, CA), making the findings ungeneralizable to the US population.

**Conclusions:**

YTH StreetConnect is a promising way to increase service utilization, provide referral access, and share resources among H/UH youth and providers. Input from H/UH youth and providers offers insights on how to improve future models of YTH StreetConnect and similar programs that assist H/UH youth.

## Introduction

About 733,000 to 2.8 million youth experience homelessness annually in the United States [1]. Homeless and unstably housed (H/UH) youth are disproportionately affected by sexual health issues, including human immunodeficiency virus/sexually transmitted diseases (HIV/STDs), pregnancy, and dating violence [2]. They are also at risk for poor mental health and underutilization of services [3]. Research suggests that linking care with H/UH youth services would improve continuity of care [4].

Nearly half of H/UH youth have no regular source of care [5], for reasons including fear of legal intervention, transportation problems, and disrespectful providers [6,7]. Since the Internet offers anonymity and accessible information, H/UH youth go online to circumvent these issues [8,9]. The majority of youth (62%) have cellphones and use them to access the Internet [10], with 85% of African American and 71% of white and Hispanic teens owning a smartphone [11]. Simultaneously, apps are becoming major tools for providers [12].

Apps have many capabilities, including tracking health status and collecting data. Apps also help with scheduling and patient interaction with providers [13]. Electronic case management has been proven to reach H/UH youth, leading to long-term care and improved health [14-16].

Accessing vital services (eg, shelter, food) is an important issue that H/UH youth face. Because health services are using technology and H/UH youth are using mobile phones and smartphones (see the app Sheltr as an example [17]), we have an opportunity to bridge this gap and increase service utilization.

With the goal of connecting H/UH youth to resources, this project sought to (1) develop an app for H/UH youth (mobile phone) and providers (tablet) in Santa Clara County, CA, USA, (2) test app usability among potential users, and (3) assess the app’s feasibility among potential users.

## Methods

Over 6 months (October 2014 to March 2015), YTH (Oakland, CA, USA) developed and tested the app YTH StreetConnect for Android and iOS mobile operating systems: StreetConnect for H/UH youth and StreetConnect PRO for providers. The project was funded by a US Centers for Disease Control and Prevention Small Business and Innovations Research grant. Phase 1 assessed the app landscape by viewing available apps and conducting expert interviews for insight on phone and tablet usage. After app development, phase 2 consisted of think-aloud usability testing among H/UH youth and providers, including an H/UH youth focus group. Both phases received exemption from the Quorum independent review board (Quorum Review, Inc, Seattle, WA, USA).

### Phase I: Formative Research and App Development

We searched the literature on H/UH mHealth programs on the databases PsycINFO, ProQuest, and EBSCO. Research terms were “H/UH youth,” “mHealth,” “mobile,” and “SMS.” We included papers dated 2010–2016 in order to highlight current trends. We categorized each paper into type of intervention: service utilization, HIV/STDs, and resource locator. We considered these three types to be essential aspects for prototype development and used them to help garner ideas for app design, look, and functionality. We then documented apps related to health, H/UH issues, and resource locators for prototype ideas. Lastly, we conducted expert interviews with providers in Santa Clara County. Based on information gathered from formative research, university students of Santa Clara University Frugal Innovation Hub developed a prototype of YTH StreetConnect, with the features outlined in Table 1.

**Table 1:** YTH StreetConnect features by app type.

YTH StreetConnect mobile app (for homeless and unstably housed youth)	YTH StreetConnect PRO tablet app (for providers)
Location-based database of services	Location-based database of services
Interactive mapping	Interactive mapping
User-submitted ratings and comments	Referral function
Emergency hotlines	Emergency hotlines
Access to sexual health information	Access to best practices
Weekly text message health tips	Medical questionnaire for clients (assesses homelessness vulnerability and sexual risk)
Accessible via Wi-Fi	Accessible via Wi-Fi

### Phase II: Usability Testing and App Refinement

We conducted think-aloud usability testing via the live app. Providers reviewed YTH StreetConnect PRO on a provided tablet, and H/UH youth reviewed YTH StreetConnect on a provided smartphone. We assessed the following questions and had participants use think-aloud usability methodology to openly state what they were doing, thinking, and feeling while using YTH StreetConnect [18]. We took notes on (1) user experience, (2) feasibility, and (3) needed changes.

After usability testing, we conducted a focus group with the same H/UH youth. The focus group was audio recorded, then transcribed with pseudonyms. Transcripts were read by 2 team members and coded through an emergent coding process, in which major themes arose from the data: app usefulness, changes needed, experience, and visuals. We considered these themes when creating the final prototype.

### Participants

We recruited participants from H/UH services in the California Bay Area. We employed one H/UH youth to promote the project at shelters. Flyers were posted at shelters, at clinics, and on craigslist (San Francisco, CA, USA).

We screened potential participants via phone for eligibility. Youth eligibility criteria were age 18–25 years, reporting an H/UH situation, and being a resident of Santa Clara County. Table 2 lists the youth participants’ demographic characteristics. The provider eligibility criterion was providing H/UH youth services, including physicians, community center leaders, health providers, and housing directors. Youth participants received a US $100 gift card for the Safeway food retailer (US $50 for usability testing, US $50 for participating in a focus group) and providers received a US $50 gift card for participation.

**Table 2:** Demographic characteristics of homeless and unstably housed youth (H/UH) participants testing the YTH StreetConnect app.

Characteristics		No.
**Sex**		
	Female	3
	Male	3
**Race/ethnicity** ^a^		
	African American	4
	Hispanic	3
	White	2
**Sexual orientation**		
	Heterosexual	5
	Not reported	1
Owned cellphone		6
Owned smartphone		5
Currently H/UH		6

^a^Race/ethnicity numbers are higher than 6 because some participants selected each race/ethnicity that applied.

## Results

### Youth Insights

#### User Experience

Overall, YTH StreetConnect was well received by H/UH youth. Youth said YTH StreetConnect was intuitive, fun, and easy to use. The phone icon allowed them to easily click and call services. They considered the map to be essential. Overall, participants thought YTH StreetConnect was a good blend of current social media and app functions:

[YTH StreetConnect] is like Google and Yelp combined...I can find what I need here really easily.

#### Feasibility

Users thought YTH StreetConnect would be helpful for H/UH youth. It was noted for its one-stop shop functions, which made it easy to access multiple resources. In addition, youth reported a high likelihood that they and other H/UH youth would use YTH StreetConnect:

*The whole app overall, it’s going to be helpful. It will help a lot of young people.*

#### Needed Changes

Participants suggested some changes, including combining the “Zip Code” and “Current Location” in one tab; adding a “home” button on all screens; and using icons to represent services. Youth said YTH StreetConnect should focus on common services (food, shelter, showers, and laundry) and that service information should be provided above map locations so that all information would be visible within a single screen.

Youth wanted other services listed, including transportation, financial and legal assistance, education, substance abuse help, food banks, and family and childcare services. These services are important to H/UH youth:

Nine times out of ten, if you’re homeless you’re probably not gonna be driving, probably gonna be on the bus...so like, you should have public transport [on YTH StreetConnect], like what bus is gonna get me there.

Participants also wanted a forum-based platform where they could share experiences. The forum would allow H/UH youth to inform peers about best services and providers, and to build community. Youth also desired a feature that would let them see the number of beds available at local shelters. Often, participants spent their only funds to travel to a shelter, only to discover that no beds were available.

**Figure 1:**
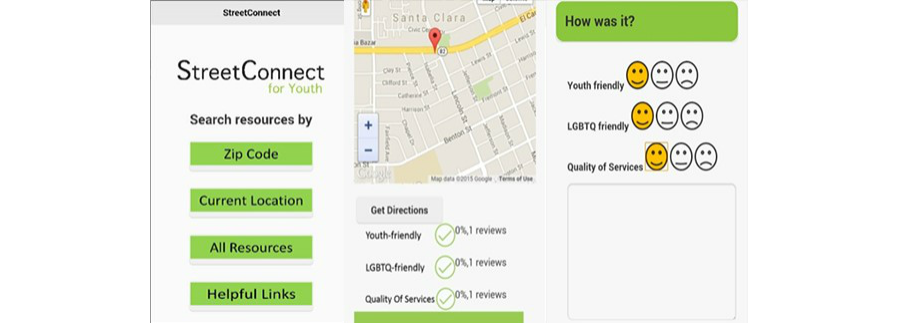
Initial prototype of YTH StreetConnect mobile app for youth.

Figure 1 shows the initial YTH StreetConnect prototype.

### Provider Insights

**Figure 2:**
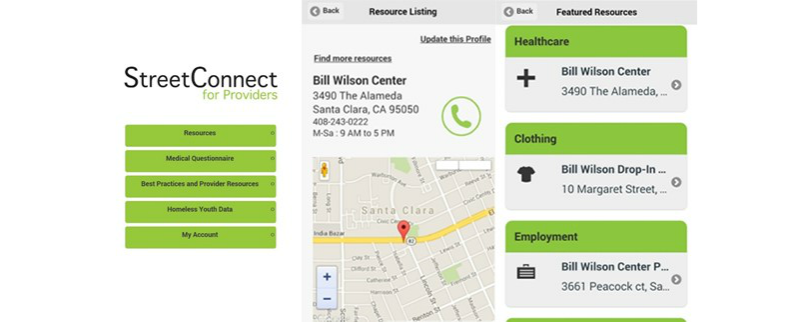
Initial prototype of the YTH StreetConnect PRO mobile app for providers.

Figure 2 shows the initial YTH StreetConnect PRO prototype.

### User Experience

Providers noted the simplicity of YTH StreetConnect and appreciated the clear images and text. Providers were able to intuitively access each function of YTH StreetConnect with little difficulty:

*Overall, [YTH StreetConnect] is nice, clean, and easy to read.*

#### Feasibility

Providers stated that YTH StreetConnect would be a helpful tool when working with H/UH youth. Providers reported that the statistics and resource information functions were most useful:

Resources is pretty much what I would need and use, and they’re definitely there.

#### Needed Changes

Providers said the medical questionnaire would provide important information and statistics on clients, but recommended to make it clear that responses would be confidential:

[The youth] will sign—but you need to tell them it’s confidential. That’s a must.

It was recommended that messages and referrals be sent via text. Providers said they usually give referrals to youth in person, but YTH StreetConnect simplified this exchange by bringing referrals online.

## Discussion

**Figure 3:**
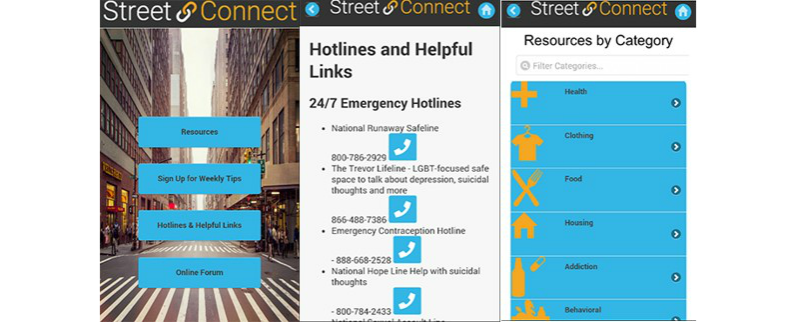
YTH StreetConnect and YTH StreetConnect PRO final apps.

YTH StreetConnect offers an accessible and appropriate way for H/UH youth and providers to locate services. We incorporated advice and suggested improvements from H/UH youth and providers in the final version (Figure 3).

We made visual enhancements to YTH StreetConnect to make it youth friendly. Implemented changes were information and map all in one screen, a peer rating system, icons for services, and an online forum. We did not implement the ability for providers to update the number of beds, largely due to limitations in funds. In addition, this feature would require extra labor for providers, who would need to update their bed availability on their own time.

YTH StreetConnect can give H/UH youth confidential access to resources, which is a central aspect of increasing service utilization among H/UH youth [19,20]. According to participants, YTH StreetConnect also provides confidential access to sexual health services, a resource that H/UH youth are more likely to use if they can access it confidentially [17]. The new online referral function for providers may also help retain H/UH youth in long-term care.

### Limitations

This prototype is for a small catchment area and we cannot predict how a national database of services will function. Future work should engage with database specialists to determine the feasibility of scaling the service up to a national database. In addition, our small sample makes it difficult to capture more in-depth experiences and feedback.

### Conclusions

We developed YTH StreetConnect and tested it with providers and H/UH youth. Because H/UH youth face challenges in service utilization, apps such as YTH StreetConnect may assist youth in finding and accessing services and improving continuity of care. YTH StreetConnect is a useful tool in streamlining services to H/UH youth online and can be especially useful as a supplement to in-person interactions between providers and youth. Future efforts could involve nonprofit agencies in conducting a national expansion of YTH StreetConnect and a pilot evaluation of its uptake among H/UH youth.

## Abbreviations

HIV/STDs: human immunodeficiency virus/sexually transmitted diseases
H/UH: homeless and unstably housed
